# Improving Classification Performance through an Advanced Ensemble Based Heterogeneous Extreme Learning Machines

**DOI:** 10.1155/2017/3405463

**Published:** 2017-05-04

**Authors:** Adnan O. M. Abuassba, Dezheng Zhang, Xiong Luo, Ahmad Shaheryar, Hazrat Ali

**Affiliations:** ^1^School of Computer and Communication Engineering, University of Science and Technology Beijing (USTB), Beijing 100083, China; ^2^Beijing Key Laboratory of Knowledge Engineering for Materials Science, Beijing 100083, China; ^3^Department of Electrical Engineering, COMSATS Institute of Information Technology Abbottabad, Abbottabad, Pakistan

## Abstract

Extreme Learning Machine (ELM) is a fast-learning algorithm for a single-hidden layer feedforward neural network (SLFN). It often has good generalization performance. However, there are chances that it might overfit the training data due to having more hidden nodes than needed. To address the generalization performance, we use a heterogeneous ensemble approach. We propose an Advanced ELM Ensemble (AELME) for classification, which includes Regularized-ELM, *L*_2_-norm-optimized ELM (ELML2), and Kernel-ELM. The ensemble is constructed by training a randomly chosen ELM classifier on a subset of training data selected through random resampling. The proposed AELM-Ensemble is evolved by employing an objective function of increasing diversity and accuracy among the final ensemble. Finally, the class label of unseen data is predicted using majority vote approach. Splitting the training data into subsets and incorporation of heterogeneous ELM classifiers result in higher prediction accuracy, better generalization, and a lower number of base classifiers, as compared to other models (Adaboost, Bagging, Dynamic ELM ensemble, data splitting ELM ensemble, and ELM ensemble). The validity of AELME is confirmed through classification on several real-world benchmark datasets.

## 1. Introduction

An ensemble learning is a machine learning process to get better prediction performance by strategically combining the predictions from multiple learning algorithms [1]. Ensembles are known to reduce the risk of selecting the wrong model by aggregating all the candidate models [2, 3].

In the process of improving ensemble accuracy and stability, different techniques have been established. These techniques vary in their approach to treat the training data, the type of algorithms used, and the combination methods followed. Bagging [4], Boosting [5], and their variants, such as Adaboost [6], are some of the popular ensembling techniques.

Traditional neural network-based classifiers usually suffer from overfitting and local optimum issues and have remained an active research subject for performance improvement by different ensemble methods. However, recently Extreme Learning Machine (ELM) has gained popularity for solving classification problems. ELM is a single-hidden layer feedforward network (SLFN) extension. Unlike the traditional classic gradient based learning algorithms, which only work for differentiable activation functions and are prone to issues like local optimum, improper learning rate, and overfitting, etc., ELM can deal with nondifferentiable activation functions and tends to reach the solution straightforward without such trivial issues [7]. Random initialization of input to hidden layer parameters in ELM helps evade the tuning process for hidden layer parameters, which extensively shortens the learning time. Although ELM is fast and achieves good generalization performance, there is still a lot of room for improvement. Several modifications have been recently introduced in the base of ELM algorithm to improve accuracy and generalization, such as optimally pruned Extreme Learning Machine (OP-ELM) [8] and Regularized-ELM [9–12].

On the contrary, ensemble learning offers an inexpensive alternative due to its performance optimization. Several approaches were proposed to generate ensembles-based ELM, such as DELM [13], EnELM [14], and DSELME [15]. Such Ensembles of Extreme Learning Machine classifiers were successful in achieving good performance for hyperspectral image classification and segmentation in a semisupervised and spatially regularized process [16]. Bagging-ELM (B-ELM) [17] is another ELM ensemble classifier, which leverages the bag of little bootstraps technique and has been found efficient for large-scale data classification. An online sequential-ELM (OS-ELM) based framework supports ensemble methods including Bagging, subspace partitioning, and cross-validating [18].

Diversity among the performance of each single classifier in the ensemble is essential for combining the predictions from several member classifiers. Different techniques are followed to introduce diversity among member classifiers. A cross-validation [13, 14] to validate each ELM before adding it to the ensemble is used. The proposed work related to ELM ensembles in the literature used a homogeneous base classifier algorithm for members' training in the model [13–15]. Motivated by the accuracy achievement of enhanced ELM algorithms and ensemble approach; we propose a heterogeneous ensemble model with different ELM algorithms for members' training. More specifically, we adopt three types of ELM algorithms, namely, Regularized-ELM [10], ELML2 [11], and Kernel-ELM [19]. These ELM algorithms are briefly described in Section 2. These base classifiers are an enhancement for the standard ELM algorithm and are chosen on the basis of their better generalization, regularization, and resilience to the outliers. A random resampling strategy is chosen to split the training data into subsets. Each member classifier is learned on a randomly chosen data subset through a randomly selected base ELM algorithm. The proposed ensemble algorithm evolves by monitoring the diversity and generalization performance of the updated ensemble during training. Majority voting method is used for combining the predictions from several member classifiers in AELME. Ten real-world benchmark datasets (Iris, Climate, Credit, Wave, Satellite, Letter, Firm, Colon, Liver, and Vowel) are used for detailed performance analysis and comparison. Experimental results, as reported in this work, show that the proposed AELME approach gives better classification accuracy on the benchmark datasets. The remainder of this paper is organized as follows: AELME ensemble algorithm and implementation details are elaborated under Section 3. Performance analysis of the proposed AELME algorithm (see Algorithm 1) is reported in Section 4, by comparing its accuracy with base classifiers {RELM, ELML2, KELM, ELM, SVM} and other ensemble methods, which include DELM, DSELME, and EnELM. Finally, the paper is concluded in Section 5. 

## 2. Background

### 2.1. The Base Classifiers

Three types of ELM classifiers, namely, ELML2, RELM, and KELM, are used as base classifiers to build AELME ensemble. Here we will briefly introduce the strengths of the selected base ELM classifiers. ELML2 [10] is a regularized algorithm-based ELM, which has all the basic ELM advantages of regression, binary, and multiclass classification. Moreover, it introduced a Lagrange multiplier based constraint optimization method. Therefore, the resultant solution is more stable and has a better generalization performance with different types of hidden nodes (feature mappings). KELM [19] is an optimization method-based Extreme Learning Machine, which links the ELM minimal weight norm property to Support Vector Machines (SVM) maximal margin for classification. It is shown that, through standard optimization for ELM, a so-called support vector network with better generalization property can be obtained by ELM Kernels. However, in comparison with standard SVM, the Kernel-ELM is less sensitive to the user-specified parameters and has fewer optimization constraints. RELM [11] is a constrained and optimized algorithm-based ELM for regression and multiclass classification. For better generalization, RELM makes a tradeoff between the structural (weight norm) and empirical risk (least square error) by regulating a proportion of them during optimization. To achieve this balance, the empirical risk in the objective function is weighted by a regulating factor gamma. For more details, the reader can refer to [10, 11, 19].

### 2.2. ELM Theory

According to the ELM theorem, the Extreme Learning Machine is built by random hidden nodes. Given a training dataset {(*x*_*i*_, *t*_*i*_)∣*x*_*i*_ ∈ *R*^*d*^, *t*_*i*_ ∈ *R*^*m*^, *i* = 1,…, *N*}, where *x*_*i*_ is the training data vector, *t*_*i*_ is the target of the training data and the number of hidden nodes (*L*). Different from other learning algorithms, ELM theory target is to reach the smallest training error with the smallest norm of the output weights [10, 20]. The minimization goal is

(1) $$\begin{array}{c} {{\left\|\beta \right\|}_{p}}^{\sigma 1}+\lambda {{\left\|H\beta -T_{\circ }\right\|}_{q}}^{\sigma 2}, \end{array}$$

where *σ*1 > 0, *σ*2 > 0, *p*, *q* = 0, (1/2), 1,2,…, +*∞* and *H* represents the output of hidden layer matrix:

(2) $$\begin{array}{c} H=\left[\begin{array}{c} h\left(x_{1}\right)\\ \vdots \\ h\left(x_{N}\right) \end{array}\right]=\left[\begin{array}{ccc} h_{1}\left(x_{1}\right) & \cdots & h_{L}\left(x_{1}\right)\\ \vdots & \ddots & \vdots \\ h_{1}\left(x_{N}\right) & \cdots & h_{L}\left(x_{N}\right) \end{array}\right]. \end{array}$$

*T*_∘_ is the target of the input data:

(3) $$\begin{array}{c} T_{\circ }=\left[\begin{array}{c} t_{1}^{T}\\ \vdots \\ t_{N}^{T} \end{array}\right]=\left[\begin{array}{ccc} t_{11} & \cdots & t_{1m}\\ \vdots & \ddots & \vdots \\ t_{N1} & \cdots & t_{Nm} \end{array}\right]. \end{array}$$

Three steps summarize ELM training algorithm [7]:

(1) Randomly assign input weights *a*_*i*_ and biases *b*_*i*_, *i* = 1,…, *L*.
(2) Compute the hidden layer output matrix.
(3) Compute the output vector:
  (4) $$\begin{array}{c} \beta =H†T_{\circ }, \end{array}$$

where *H*† represents Moore–Penrose (MP) generalized inverse of matrix *H* and *T*_∘_ = [*t*_1_,…,*t*_*N*_]^*T*^.

To compute MP inverse by applying the orthogonal projection, *H*† = (*H*^*T*^*H*)^−1^*H*^*T*^, if *H*^*T*^*H* is nonsingular. A positive value (1/*λ*) is added to the diagonal of *H*^*T*^*H* or *HH*^*T*^ in the calculation of the output weights *β* as ridge regression theory stated. At the end, we have a solution which is equivalent to the ELM optimization solution [7, 19], with *σ*1 = *σ*2 = 2, which is more stable and has better generalization performance. So, to enhance the stability of ELM, *β* and the output function are computed, respectively, by

(5) β=HT1λ+HHT−1fx=hxβ=hxHT1λ+HHTT.

Given the above-mentioned advantages of ELM, we propose using it in ensemble to achieve better classification results. Naturally, there is nothing to gain by combining identical models while doing precisely the same actions. Consequently, the base classifiers must commit their errors on different instances, which is the informal meaning of the term diversity. We use three variants of ELM to improve diversity among the base classifiers. Overall, the proposed ensemble is designed to improve performance in terms of accuracy and it is more stable.

## 3. Advanced Ensemble for Classification Using Extreme Learning Machines

**Figure 1:**
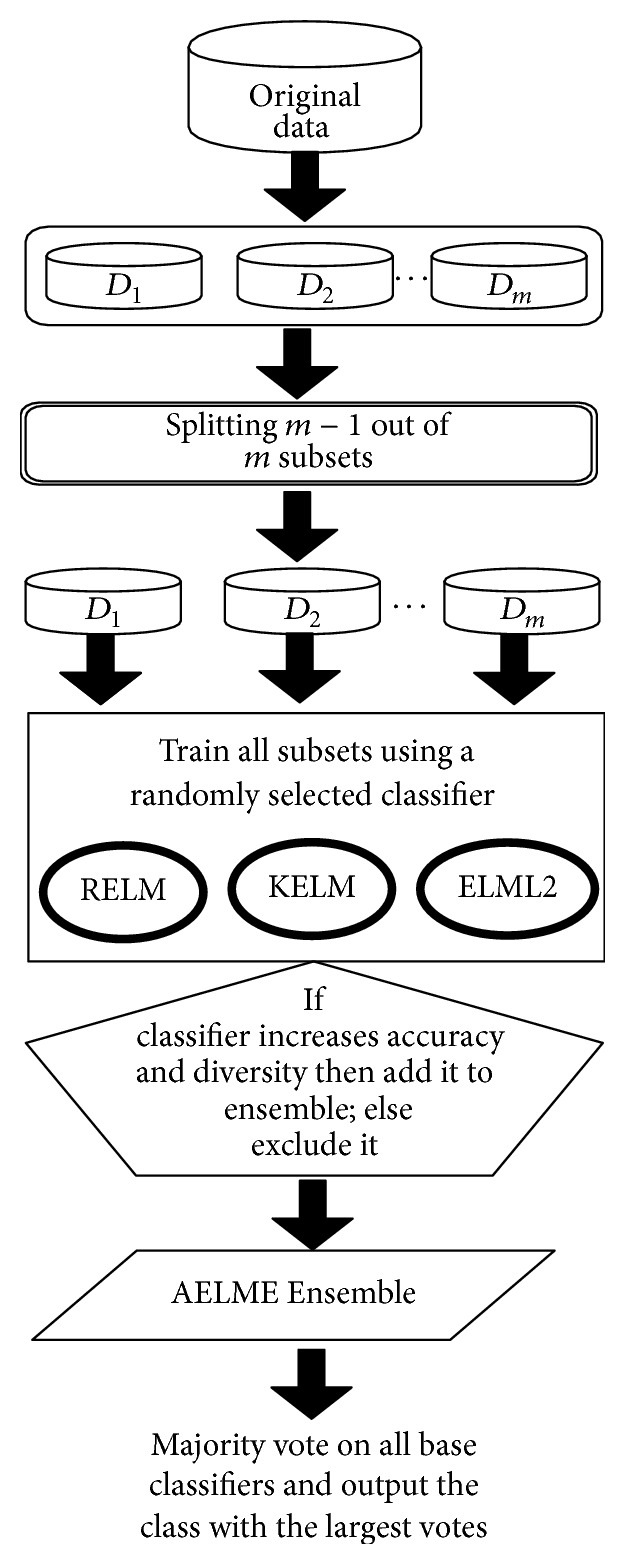
The general scheme of the proposed AELME.

Unlike designing a single classifier in traditional pattern recognition field, ensemble learning aims at constructing multiple diverse classifiers and combines their outputs to form a hybrid predictive model. Consequently, the overall classification performance of ensemble classifier tends to be better than when using a single classifier. As ELM uses random weights, it often has a low misclassification rate. To improve the classification rate performance, a number of multiclassifiers based on ensemble learning have been proposed in [21, 22]. In this work, we use data splitting of training data and three types of ELM algorithms as the base learners to build a classifier on split data and majority voting to combine outputs from all member classifiers in ensemble pool. Different training parameters of base ELM learning algorithms allow each member classifier to generate different decision boundaries. Hence different errors are made resulting in a reduced overall error for the ensemble. Training data distribution has an effect on the generalization of the learning classifier. For example, a training set may contain instances from a particular class such that the feature values of those instances are skewed towards a particular intraclass member. To address this issue, we divide training dataset into different parts as it tends to preserve the original data distribution by using random resampling on the dataset. Consequently, classifiers with large diversity and different errors are produced. For example, if we divide training data *D* into 3 parts *D* = {*D*_1_, *D*_2_, *D*_3_} then we have three training subsets: {*D*_2_, *D*_3_}, {*D*_1_, *D*_3_}, and {*D*_1_, *D*_2_}. A sufficient and necessary condition for the ensemble to outperform its base members is that component learners should be simultaneously accurate and diverse; therefore, a new member is added to AELME if it increases both diversity (in terms of disagreement) and accuracy of the model. General description of the model is shown as flowchart in Figure 1.

### 3.1. Architecture

The training dataset is divided randomly into *m* equal size subsets. If we have *N* samples, then the size of each subset will be (*N*/*m*). To maximize the diversity among reconstructed training datasets, each new training set is obtained through resampling on *m* − 1 out of *m* subsets. Then, training with each subset is done using one out of the three base classifier learners, which is selected randomly. The trained classifier is added to the ensemble and the process is repeated for all the remaining subsets. In the next iteration, if diversity and accuracy of the current ensemble *E* are improved with the addition of *E*_*K*_ (ensemble number *K*), then it will be retained in the updated ensemble and excluded otherwise. The final ensemble model is a mixture of all classifiers trained on all subsets. Our model has three types of ELM algorithms, specifically Regularized-ELM, ELML2, and Kernel-ELM. Once the training is complete, labels for tested data are obtained by majority voting method applied to the member classifiers' outputs in the evolved ensemble.

### 3.2. Testing Stage

#### 3.2.1. Majority Vote

The implementation procedure for the ensemble construction and training stage is described in the algorithm of AELME.

Given a testing instance (*X*, *t*), an ensemble of *m* × *K* predictors is created. For pattern *X*, we use majority voting to make the final decision. Suppose we have one *C*-class's problem. If the *k*th ELM in the ensemble predicts the pattern *X* as class *C*, we assign vote one to it and zero otherwise. Once all the votes have been assigned, the class that receives the highest votes from all predictors is considered the predicted class.

#### 3.2.2. Weighted Sum

Given a testing instance (*X*, *t*), an ensemble of *m* × *K* predictors are created. In decision making on the ensemble, for pattern *X*, we use weighted sum to make the final decision. Suppose that there is *C*-classe's problem, and we calculate the weighted sum for all classifiers for all classes. The class that receives the maximum weighted sum from all predictors is considered as the predicted class:

(6) $$\begin{array}{c} C\left(\;y\;\right)=arg\;max\overset{m\times K}{\underset{j=1}{\sum }}\alpha_{j}\cdot \left(\;f_{j}\left(\;y\;\right)=C\;\right), \end{array}$$

where *α*_*j*_ is the weight of base learner and *f*_*j*_(*y*) is the prediction result.

## 4. Simulation and Discussion

### 4.1. Simulation Settings

To test the performance of the model, we carry out our simulation experiments on ten diverse datasets from several domains with different characteristics and diversity in size and input feature dimensions. The datasets come from machine learning repository (UCI) [23] besides including one dataset from LIBSVM [24] which is sourced in [25]. A brief description of the datasets is included in Table 1; more details of what characterizes the problem domains of the datasets can be found on the web pages of those repositories. The simulations of different algorithms on all the datasets are carried out in MATLAB 8.1.0 environment running on Intel® Core i5, 2.4 GHZ CPU with 4 GB RAM. To remove any bias from the results, we repeat the experiment 10 times and calculate the average accuracy for all iterations. Training data is split into 2–8 equal size subsets (according to the number of instances in the datasets) using random resampling. For a fair evaluation, we use the same split number of subsets for all ensembles.

### 4.2. User-Specified Parameters

**Figure 2:**
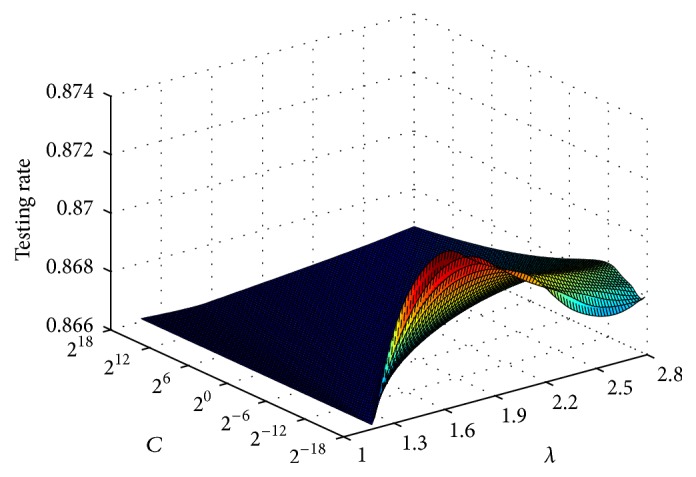
Surface plot in terms of performance of AELME sensitivity to the user-specified parameters (*C*, *λ*): an example on Wave dataset.

To achieve good generalization performance, the cost parameter *C* and the Kernel parameter *λ* of the base ELM classifiers (Regularized-ELM, ELML2, and Kernel-ELM) need to be chosen appropriately. We use search grid over *C* and *λ* to determine optimal values. For each dataset, we have used different values of C and different values of *λ*. The range of *λ* is {0.1,0.2,…, 10} and the range of *C* is {2^−50^, 2^−48^,…, 2^50^}. The number of hidden nodes is selected from the range {10,20,…, 1000}. Optimal values of the selected parameters are shown in Table 2. To study the generalization performance of AELME on the combination of (*C*, *λ*), we select a medium dataset size (Wave). From Figure 2, it can be noticed that changing the value of *C* and (*λ*) parameters does not have a significant effect on the accuracy. So, the model seems to have less sensitivity towards the combination parameters (*C*, (*λ*)).

### 4.3. Metrics

We use a set of measures to evaluate the efficiency of AELME model. We use accuracy as an indication of the classification output correctness. Standard deviation of the accuracy rates is used as an indication of ensemble stability; the lower standard deviation the method has, the more stable the method is.

The cost of training a new (test) data should not have a significant change on the ensemble accuracy when we train the ensemble with any training set of size a bit more or less than the original data. We use the decrease or increase in average absolute error averaged over all our datasets, assuming they represent a reasonable real-world distribution of datasets. The average relative error reduction measure is also used. For two algorithms [26] A and B with errors *e*1 and *e*2, the decrease in relative error between A and B is (*e*1 − *e*2)/*e*1. The average relative error is the average (over all our datasets) of the relative error between the pair of algorithms compared. We compared our model with all other approaches. A negative value for the error implies that our model reduces error, while positive values correspond to increase in error for our model. Time costs of Adaboost, Bagging, EnELM [14], DSELME [15], DELM [10], and AELME are also compared.

### 4.4. Diversity Measures

It is not straightforward to express actual diversity among classifiers in an ensemble through standard diversity measure. While there are some measures with which to approximate its value, there is no perfect one [27, 28]. Here, we use disagreement to measure diversity and also *Q*-Statistic which is recommended in [29].

#### 4.4.1. Disagreement

The diversity within the whole ensemble is calculated by averaging disagreement measure [30] over all pairs of base classifiers:

(7) $$\begin{array}{c} Dis=\overset{L}{\underset{i=1}{\sum }}\overset{L}{\underset{k=j+1}{\sum }}dis_{j,k}, \end{array}$$

where *L* is the number of base classifiers, dis_*j*,*k*_ is the disagreement between classifier *j* and classifier *k*. This measure is defined based on the intuition that two diverse classifiers perform differently on the same training data. Disagreement measure is used to test the diversity within the whole set of base classifiers. The diversity increases with the value of the disagreement measure.

#### 4.4.2. *Q*-Statistic

Yule's *Q*-Statistic [31] measures the similarity between two classifiers (*C*_*i*_ and *C*_*j*_). It can be calculated as follows:

(8) $$\begin{array}{c} Q_{i,j}=\frac{a*b-c*d}{a*b+c*d}, \end{array}$$

where (*a*, *b*) represents the number of samples for which both the classifiers are making (correct, wrong) classification, respectively. Similarly, (*c*, *d*) represents the number of samples for which both the classifiers are committing errors. Then the averaged *Q*_*m*_ value for more than two classifiers can be calculated as follows:

(9) $$\begin{array}{c} Q_{m}=\frac{2}{C\left(C-1\right)}\overset{C-1}{\underset{i=1}{\sum }}\overset{C}{\underset{j=i+1}{\sum }}Q_{ij}, \end{array}$$

where *C* is the number of classifiers and *Q*_*m*_ ∈ [−1,1]. When *Q*_*m*_ equals zero, it implies that the classifiers are independent. And if *Q*_*m*_ equals one, it implies identical (dependent) classifiers. A positive value of *Q*_*m*_ means that the classifiers have classified the same input correctly and negative value of *Q*_*m*_ means that the classifiers have committed errors on different inputs. Diversity increases if *Q*_*m*_ value decreases and vice versa. However, it is not easy to attain large negative *Q*_*m*_ value [29] for more than two classifiers. For *Q*_*m*_ calculations, we use the diversity measure toolbox (http://pages.bangor.ac.uk/~mas00a/ensemble_diversity.html).

### 4.5. Statistical Tests

#### 4.5.1. Wilcoxon Test

The Wilcoxon test is a nonparametric statistical test [32]. The purpose is to compare between two models and several data samples to measure the difference between them and to know if one is significant or both are equal. It is insensitive to the sample size and outliers. Our null hypothesis (*H*_0_) is that “there is no difference between our model and the one to which it is being compared.” The alternative hypothesis (*H*_1_) is that our model is more significant than the compared model. We use a significance level of 95% (threshold is equal to 0.05). Small values of *p* (*p* value) cast doubt on the verity of the null hypothesis. A small *p* value verifies that one approach is more significant than the other. The procedure is as follows: find the performance (Pf) difference between the two compared algorithms. Rank the absolute values of Pf in ascending order (the smallest value = 1, the second value = 2, and so on). If there are two equal values, then assign the average rank for all equal values. Compute the negative and the positive rank sum according to the Pf sign. Find the minimum of the two sums (Wilcoxon statistic: *W*). Find the critical value of *W* [33] that corresponds to the dataset number with a level of significance used to examine if the null hypothesis can be rejected. For more details, the reader can refer to [34].

#### 4.5.2. Friedman Test

Friedman test is a nonparametric statistical test [35]. The purpose is to compare the performance of multiple models and several data samples to measure the differences between them and to determine whether there is a significant difference or they are equal. Our null hypothesis (*H*_0_) is that there is no difference between AELME and other algorithms. The alternative hypothesis (*H*_1_) is that there is a significant difference between at least two of the compared models. The significance level used is 95% (threshold is equal to 0.05).

### 4.6. Statistical Results

The (*p* value) result of Wilcoxon test of AELME compared with all algorithms is shown in Table 5. It is less than 0.05 in all cases; that implies the null hypothesis (*H*_0_) is rejected, and the alternative hypothesis (*H*_1_) is accepted. There is enough evidence that our model is more significant as compared to other models reported in this work. Moreover, the result of Friedman test value is 22.08, with *p* value that equals 0.0086. We reject the null hypothesis and accept the alternative hypothesis that there are differences between the compared models.

### 4.7. *Q*-Statistic Experiments

We use Wave, Liver, and Satellite datasets to do experiments; we do not prespecify the individual accuracy nor the individual dependency. Wave data is three-class data; we divide training data into subsets of 4, 4, and 2 features (500 parts). Then train three classifiers {RELM, KELM, ELML2} one on each subset of features. The Liver is two-class data with 6 dimensions. Satellite is a seven-class data with 10 dimensions. We split data randomly into training/testing subsets. The training/test sets split is 470/230 for Wave data, 230/115 for Liver data, and 800/400 for Satellite data. We generate 2500 ensembles for all data. The setting of our experiments is shown in Table 8. We observe from experiments a general trend of improvement in accuracy towards low values of *Q*. In all experiments, the maximum improvement corresponds to negative values of *Q* as shown in Table 9. However, at the same time there exists a range of improvements against these *Q* values, while the top-improvements are dispersed across a wide spectrum of negative *Q*-values. Almost all ensembles in our experiments show accuracy improvement over the single best classifier (Ensemble Accuracy − Maximum individual accuracy). Nevertheless, we cannot draw a conclusion that there is strong relationship between accuracy and diversity for all ensembles, because it depends on the experiment settings. Moreover, there is a need for more dedicated, in-depth research to investigate the relationship between accuracy and diversity.

### 4.8. Performance Analysis and Discussion

The classification experiments on datasets are performed using Bagging, Adaboost, Regularized-ELM (RELM) [10], ELML2 [11], Kernel-ELM (ELMK) [19], EnELM [14], DELM [13], DSELME [15], and AELME algorithms. The average classification accuracy rates with their corresponding standard deviations of the experiments over ten runs are shown in Table 4. Accuracy rates on the tested datasets show the strength of the model, as we can observe from results that our model achieves the highest accuracy rates in most cases. The base classifiers of our model ELML2, Regularized-ELM, and Kernel-ELM have accuracy rates less than the ensemble model. From Table 4 we observe that the accuracy rates on almost all datasets of Bagging and Adaboost algorithms are lower than our model and they have low accuracy rates on Wave, Credit, and Firm datasets. Moreover, we use weighted sum method in all base classifiers to test AELME on unseen data.  Table 6 shows the accuracy rates using weighted sum. We observe from results weighted sum method outperforms majority vote in most datasets. Stability is an important factor related to whether the ensemble classifier can improve the accuracy rate of classification. To analyze the stability of AELME, we repeat the experiments for 10 times on Climate dataset. 200 + 8, 200 + 16, 200 + 32, 200 + 64, and 200 + 128 instances are selected in sequence, corresponding to 1st to the 5th group, respectively. The standard deviation of the accuracy rates is calculated based on these 10 runs. Stable classifiers are less likely to overfit. To make use of the variations of the training set, the base classifier should be unstable [35]; otherwise, the resultant ensemble will be a collection of almost identical classifiers. As shown in Table 7, our ensemble classifier is more stable than all the base classifiers. Disagreement is a measure of diversity. As shown in Table 3, it is mostly increased as the size of the dataset increases. This demonstrates that diversity is increased between base classifiers in AELME.

**Figure 3:**
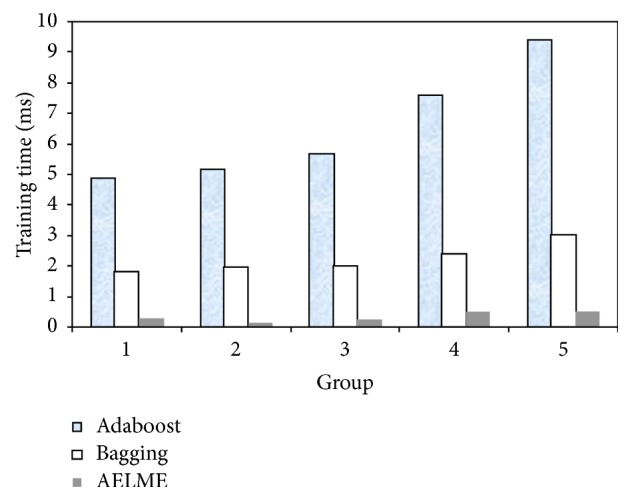
Training time of Adaboost, Bagging, and AELME.

The mean average error (MAE) of our model is the lowest one among all algorithms on all the datasets as shown in Table 10. There is a relative error reduction of our model compared to almost all other ensembles tested on all the datasets in this research. For example, for Letter dataset, there is an error reduction of 147% compared to Bagging, 157% compared to Adaboost, 175% compared to EnELM, 54% compared to DSELME, and 171% compared to DELM. The average absolute error of our model is 0.2758 which is the smallest among all ensembles as shown in Table 10. There is an average error reduction of 39% for DELM, 43% for DSELME, 35% for EnELM, 44% for Adaboost, and 59% for Bagging. We compare the time costs of Bagging, Adaboost, and AELME algorithms. 200 + 8, 200 + 16, 200 + 32, 200 + 64, and 200 + 128 instances are selected in sequence, corresponding to 1st to the 5th group, respectively, as shown in Figure 3. For Climate dataset, we observe that Adaboost algorithm is the most time-consuming algorithm, and the time cost of the AELME algorithm is less than the Bagging algorithm and Adaboost. The average training time of all algorithms is compared by taking the average of ten runs of the ten datasets.  Table 11 shows average training time for the different algorithms. We observe that our algorithm training time on some datasets is higher than other ensembles due to the computations in our algorithm.

## 5. Conclusion

In this work, we have discussed an advanced approach for classification using different ELM algorithms, namely, Regularized-ELM, ELML2, and Kernel-ELM. Each learner member is independent of the other to achieve diversity within the proposed ELM ensemble (AELME). By using different types of ELM algorithms in the ensemble and by using different training datasets for each classifier, it allows the base classifiers to generate different decision boundaries and different errors while reducing the total error. So, a combination of all the classifiers achieves better classification accuracy and the generalization performance of the ensemble increases. Experimental results show that the proposed AELME model is accurate and stable and outperforms other models. It would be an interesting future work to identify the optimal number of classifiers to be used in an ensemble for improving overall accuracy. Furthermore, in the future we will discuss the applications of the proposed method in some practical fields, for example, the Internet of Things and cyberphysical systems [36, 37].

## Figures and Tables

**Algorithm 1:**
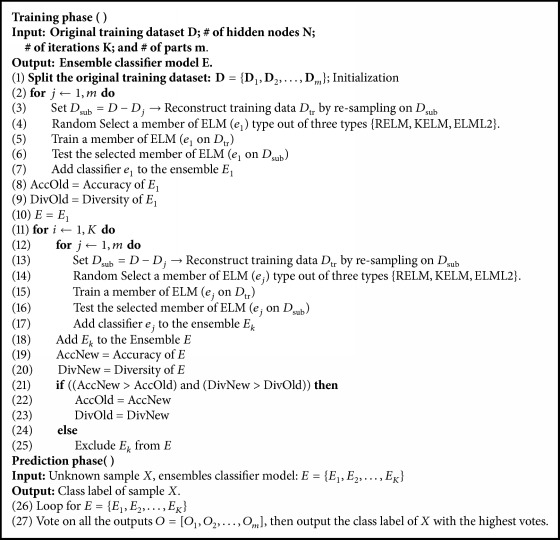
AELME.

**Table 1:** Datasets used in the experiments.

Dataset	Number of Instances	No. of Attributes	No. of Classes
Iris	150	4	3
Climate	540	18	2
Credit	690	15	3
Wave	5000	21	3
Satellite	6435	36	7
Firm	10800	20	4
Letter	20000	17	26
Colon	62	2000	2
Liver	345	6	2
Vowel	990	10	11

**Table 2:** Optimal values of ELMs' parameters (nh: number of hidden nodes).

Dataset	*C*	*λ*	nh
Iris	2^30^	1.2	20
Climate	2^20^	1.2	20
Credit	2^10^	1.2	20
Wave	2^32^	1.2	310
Satellite	2^32^	1.4	310
Firm	2^32^	1.2	310
Letter	2^32^	1.2	700
Colon	2^−16^	0.3	80
Liver	2^30^	1.2	20
Vowel	2^30^	1.2	200

**Table 3:** Disagreement measurement between base classifiers in AELME.

Dataset	Disagreement
Iris	0.0169
Climate	0.0530
Credit	0.0946
Wave	0.1083
Satellite	0.0568
Firm	0.0751
Letter	0.1607
Colon	0.0500
Liver	0.0289
Vowel	0.0130

**Table 4:** Comparisons of the average accuracy rates with their corresponding standard deviations of all the algorithms on the datasets.

Dataset	AELME	SVM	ELML2	RELM	ELMK	Bagging	DELM	EnELM	DSELME	Adaboost	ELM
Iris	0.9906 ± 0.0132	0.9670 ± 0.7100	0.9873 ± 0.0287	0.9901 ± 0.0338	1 ± 0.00	0.9656 ± 0.0099	0.9625 ± 0.00	0.9688 ± 0.0211	0.9500 ± 0.1234	0.9750 ± 0.0221	0.9856 ± 0.0151
Climate	0.9100 ± 0.0091	0.9000 ± 0.4470	0.8967 ± 0.0111	0.8933 ± 0.0064	0.9000 ± 0.00	0.9000 ± 0.0163	0.8707 ± 0.0158	0.8640 ± 0.0176	0.8920 ± 0.0250	0.9000 ± 0.0761	0.8953 ± 0.0095
Credit	0.7569 ± 0.0047	0.7500 ± 1.203	0.7495 ± 0.0094	0.7422 ± 0.0106	0.7426 ± 0.00	0.7245 ± 0.0237	0.7485 ± 0.0089	0.74657 ± 0.0143	0.7451 ± 0.0122	0.7034 ± 0.0280	0.7441 ± 0.0109
Wave	0.8674 ± 0.0022	0.8510 ± 1.68	0.8563 ± 0.0345	0.8495 ± 0.0323	0.8589 ± 0.00	0.8109 ± 0.0070	0.8712 ± 0.0026	0.8682 ± 0.0049	0.8628 ± 0.0051	0.7341 ± 0.0036	0.8587 ± 0.0245
Satellite	0.9017 ± 0.0051	0.8803 ± 2.176	0.8816 ± 0.0383	0.8903 ± 0.0345	0.8204 ± 0.00	0.8360 ± 0.0031	0.8593 ± 0.0014	0.8789 ± 0.0179	0.8454 ± 0.0017	0.8235 ± 0.0051	0.8595 ± 0.0399
Firm	0.9000 ± 0.00072	0.8888 ± 1.12	0.88244 ± 0.0219	0.85441 ± 0.0583	0.9001 ± 0.00	0.6873 ± 0.0060	0.8993 ± 0.0021	0.893 ± 0.0025	0.89445 ± 0.0019	0.8211 ± 0.0026	0.8831 ± 0.0414
Letter	0.9353 ± 0.0133	0.9237 ± 0.26	0.8103 ± 0.3265	0.9300 ± 0.2538	0.9315 ± 0.00	0.8400 ± 0.0085	0.8246 ± 0.0011	0.8221 ± 0.0016	0.9006 ± 0.0022	0.8338 ± 0.0074	0.8200 ± 0.9287
Colon	0.8900 ± 0.0211	0.8438 ± 0.00	0.8500 ± 0.0707	0.8400 ± 0.1137	0.8000 ± 0.00	0.85000 ± 0.0082	0.8450 ± 0.0158	0.8350 ± 0.0474	0.8600 ± 0.0211	0.8700 ± 0.0211	0.8200 ± 0.0882
Liver	0.7420 ± 0.0155	0.6900 ± 2.6	0.6580 ± 0.0454	0.6730 ± 0.0497	0.7200 ± 0.00	0.7000 ± 0.0194	0.6950 ± 0.0165	0.6540 ± 0.0433	0.6630 ± 0.0350	0.7000 ± 0.0236	0.6666 ± 0.0506
Vowel	0.6620 ± 0.0183	0.5660 ± 3.24	0.5120 ± 0.0488	0.5810 ± 0.0842	0.6380 ± 0.00	0.6700 ± 0.1180	0.6333 ± 0.00213	0.5680 ± 0.0306	0.5580 ± 0.0473	0.6673 ± 0.0374	0.5860 ± 0.0400

**Table 5:** Wilcoxon signed rank statistical test result of AELME versus all the algorithms upon all data sets. *p* values are small which implies the significance of the AELME approach as compared to the other algorithms (here Alg means Algorithm).

AELME versus Alg	*p* value
ELML2	0.0009770
RELM	0.0009766
ELMK	0.0097000
Bagging	0.0019500
DELM	0.0029200
EnELM	0.0019500
DSELME	0.0009766
Adaboost	0.0019500
ELM	0.0009765
SVM	0.0009760

**Table 6:** Accuracy rates of AELME upon the datasets using weighted sum method.

Dataset	Accuracy
Iris	0.9930
Climate	0.9067
Credit	0.7696
Wave	0.8748
Satellite	0.898
Firm	0.9008
Letter	0.9058
Colon	0.9
Liver	0.74
Vowel	0.71

**Table 7:** Standard deviation of accuracy rates of AELME and base classifiers {ELM, ELMR, ELML2} upon Climate dataset. 200 + 8, 200 + 16, 200 + 32, 200 + 64, and 200 + 128 instances are selected in sequence, corresponding to 1st to the 5th group, respectively.

Group	AELME	ELM	ELMR	ELML2
1st	0.034577	0.116800	0.081347	0.110030
2nd	0.025810	0.098455	0.153540	0.0769327
3rd	0.044799	0.126870	0.085248	0.096722
4th	0.021785	0.048463	0.056345	0.039907
5th	0.026814	0.055462	0.050303	0.062163

**Table 8:** Description of *Q*-Statistic experiments. (*N* is number of samples, #Att. is number of attributes, #Class is number of classes, and #Ens. is number of ensembles).

Dataset	*N*	#Att.	#Class	#Ens.
Liver	345	6	2	700
Satellite	1200	10	7	1300

*Feature subspace method*				
Dataset	Wave			
*N*	700			
#Att.	10			
#Class	3			
#Ens.	500			
The set of 10 features was divided into permutations subsets (first 500 permutations) of 4, 4, and 2.				

**Table 9:** Maximum improvement (Max-Impr) of ensemble accuracy over the single best classifier (Ensemble Accuracy − Maximum individual accuracy) with their corresponding *Q*-values.

Dataset	Max-Impr	*Q*
Liver	0.320	−0.8924
Wave	0.175	−0.0600
Satellite	0.498	−0.4000

**Table 10:** Mean Absolute Error (MAE), Relative Reduction Error (RErRed) and their Averages (AvgAbsEr, AvgRErRed, resp.), and Standard Deviation (STD) of all Algorithms.

Dataset	Measure	AELME	DELM	DSELME	EnELM	Adaboost	Bagging
Iris	MAE	0.0094	0.0375	0.05	0.0312	0.025	0.0344
	RErRed (%)		−299	−432	−232	−166	−266
	STD	0.0132	0	0.1234	0.0211	0.0221	0.0099

Climate	MAE	0.09000	0.1293	0.1080	0.1360	0.1000	0.1000
	RErRed (%)		−44	−20	−51	−11	−11
	STD	0.0091	0.0158	0.0250	0.0176	0.0761	0.0163

Credit	MAE	0.2431	0.2515	0.2549	0.2534	0.2966	0.2755
	RErRed (%)		−3	−5	−4	−22	−13
	STD	0.0047	0.0089	0.0122	0.0143	0.0280	0.0237

Wave	MAE	0.1326	0.1288	0.1372	0.1318	0.2659	0.1891
	RErRed (%)		3	−3	1	−101	−43
	STD	0.0022	0.0026	0.0051	0.0049	0.0036	0.0070

Satellite	MAE	0.0983	0.1407	0.1546	0.1211	0.1765	0.1640
	RErRed (%)		−43	−57	−23	−80	−67
	STD	0.0051	0.0014	0.0017	0.0179	0.0051	0.0031

Firm	MAE	0.1000	0.1007	0.1056	0.1070	0.1789	0.3127
	RErRed (%)		−1	−6	−7	−79	−213
	STD	0.00072	0.0021	0.0019	0.0025	0.0026	0.0060

Letter	MAE	0.0647	0.1754	0.0994	0.1779	0.1662	0.1600
	RErRed (%)		−171	−54	−175	−157	−147
	STD	0.0133	0.0011	0.0022	0.0016	0.0074	0.0085

Colon	MAE	0.8500	0.1550	0.1400	0.1650	0.1300	0.1500
	RErRed (%)		82	84	81	85	82
	STD	0.0211	0.0158	0.0211	0.0474	0.0211	0.0082

Liver	MAE	0.6580	0.3050	0.3370	0.3460	0.3000	0.3000
	RErRed (%)		54	49	47	54	54
	STD	0.0155	0.0165	0.0350	0.0433	0.0236	0.0194

Vowel	MAE	0.5120	0.3667	0.4420	0.4320	0.3327	0.3300
	RErRed (%)		28	14	16	35	36
	STD	0.0183	0.0213	0.0473	0.0306	0.0374	0.1180

	AvgAbsEr	0.2758	0.1791	0.1829	0.1901	0.1972	0.2016
	AvgRErRed		− 39	− 43	− 35	− 44	−59

**Table 11:** Average training time (×10^3^ s) of the datasets in seconds.

Dataset	AELME	EnELM	DSELME	DELM	Ada	Bag	ELM	RELM	ELML2	ELMK
Iris	0.00004	0.0008	0.00046	0.0003	0.00012	0.00106	0.000025	0.000036	0.00005	0.000013
Climate	0.0002	0.0022	0.00126	0.0018	0.00404	0.03736	0.000052	0.000204	0.00005	0.000095
Credit	0.0008	0.0044	0.00225	0.002	0.01219	0.10610	0.000047	0.001170	0.00007	0.000134
Wave	0.138	0.028	0.008	0.0107	0.1329307	1.09000	0.000222	0.001234	0.00028	0.004964
Satellite	0.1458	0.032	0.017	0.026	0.36782	2.30770	0.000304	0.210789	0.00038	0.012488
Firm	0.182	0.045	0.0393	0.11487	3.98310	3.9797	0.000373	0.401937	0.00046	0.019656
Letter	0.878	0.229	0.287	0.2911	6.68	6.88	0.000638	1.238600	0.00075	0.066160
Colon	0.000148	0.000808	0.000148	0.003449	0.000096	0.000131	0.000033	0.000080	0.000069	0.000005
Liver	0.000142	0.0001045	0.000509	0.002902	0.012979	0.0130564	0.000027	0.000076	0.0000421	0.000039
Vowel	0.0007082	0.0004571	0.0016411	0.0029796	0.1480	0.151	0.000075	0.001246	0.0000764	0.000184
